# Applicability of the Electro-Vectorcardiogram in Current Clinical Practice

**DOI:** 10.5935/abc.20190095

**Published:** 2019-07

**Authors:** Carlos Alberto Pastore, Nelson Samesima, Horacio Gomes Pereira Filho, Nancy Maria Martins de Oliveira Tobias, Bruna Affonso Madaloso, Mirella Esmanhotto Facin

**Affiliations:** Instituto do Coração (InCor) do Hospital das Clínicas da Faculdade de Medicina da Universidade de São Paulo (FMUSP), São Paulo, SP - Brazil

**Keywords:** Electrocardiography/methods, Vectocardiography/methods, Electrophysiological Phenomena, Internal Medicine/methods

## Abstract

The electrocardiogram (ECG) has been reinvigorated by the identification of electrical alterations that were not definitely clarified before. In this context, and mainly regarding the definition of arrhythmogenic substrates, the association of the ECG with the vectorcardiogram (VCG) has gathered much more information about the cardiac electrical phenomena, thus allowing us to differentiate potentially fatal cases from benign ones. Obtaining a VCG concomitantly with the performance of an ECG has led to a significant gain in the definition of extremely sophisticated pathologies, which function suffer some type of structural or dynamic alterations, involving either the reduction or enhancement of ionic channels and currents.

The classic aspects of the ECG/VCG association in the differential diagnosis of myocardial infarctions, conduction disorders, atrial and ventricular hypertrophies, and the correlations between these electrical disorders are still valid and assertive. The association of these pathologies is further clarified when they are seen through the ECG/VCG dyad.

The three-dimensional spatial orientation of both the atrial and the ventricular activity provides a far more complete observation tool than the ECG linear form. The modern analysis of the ECG and its respective VCG, simultaneously obtained by the recent technique called electro-vectorcardiogram (ECG/VCG), brought a significant gain for the differential diagnosis of some pathologies. Therefore, we illustrate how this type of analysis can elucidate some of the most important diagnoses found in our daily clinical practice as cardiologists.

## Introduction

The study of vectorcardiography began during the 1940's and publications reached a peak between the 1960's and 1970's, correlating this method with the heart diseases best known at that time. The great difficulty then was linked to the fact that the VCG device could not be easily moved around. The images were thus not immediately obtained, and so the vectorcardiogram was a tool to be used *a posteriori* to resolve doubts about specific electrocardiograms in some special situations. Additionally, the advent of echocardiography and its later improvement, as well as the emergence of computed tomography and magnetic resonance imaging, led to a decreased use of both the electrocardiogram and, mainly, of the vectorcardiogram from the 1980's until the end of the 1990's. This temporal gap, associated to a diminished interest in electrovectorcardiography, resulted in a significant decrease in the number of cardiology centers capable of performing and interpreting a vectorcardiogram.

However, with the development of invasive electrophysiology (electroanatomic mapping), genetics and molecular biology, many electrical conditions have been unveiled, resulting in the identification of their clinical/electrocardiographic patterns, since such conditions can lead to sudden death.^1-7^

The technological developments seen during the 1990s also affected electrovectorcardiography. The sophistication brought by the use of computers, algorithmic systems and Fourier transforms allowed us to obtain vectorcardiographic information in a much simpler and quicker form, in color, and as three-dimensional images.

The electrocardiogram (ECG) was therefore reinvigorated by the identification of electrical alterations that had not yet been definitely observed, so that from the year 2000 until the present decade an increasing number of publications related to electrovectorcardiography has been observed. In this context, and mainly regarding the definition of arrhythmogenic substrates, it was observed that the association of the ECG and the vectorcardiogram (VCG) methods could provide much more information about the cardiac electrical phenomena, thus increasing its employment and allowing us to differentiate potentially fatal cases from benign ones.^8-10^

We now find ourselves in a moment where the performance and reading of the vectorcardiogram is carried out in just a few specific centers around the world. Moreover, our team is involved with the teaching of electrocardiography to undergraduate and postgraduate students in the medical area. Therefore, we feel there is an urgent need to teach vectorcardiography, considering that its spatial visualization of the cardiac electrical activation makes it a lot easier to understand and memorize the basic and more complex electrocardiographic notions.^11^

Obtaining a VCG concomitantly with the performance of an ECG has led to a significant gain in the definition of extremely sophisticated pathologies, of which genetic mutations cause their function to suffer some type of structural or dynamic alterations, involving either the reduction or enhancement of ionic channels and currents.

**Figure 1:**
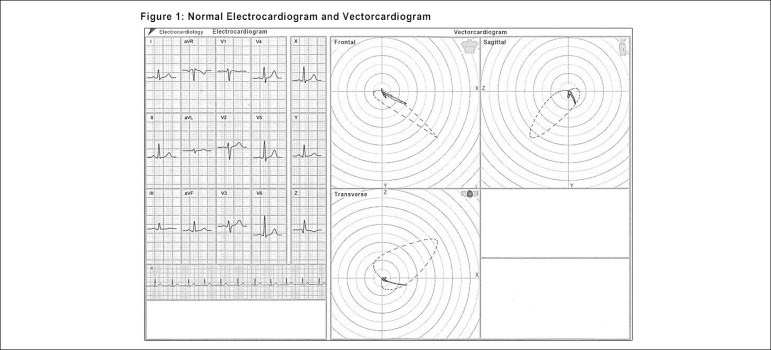
Electrocardiogram vs. Vectorcardiogram.

The classic aspects of the ECG/VCG association in the differential diagnosis of myocardial infarctions, conduction disorders, atrial and ventricular hypertrophies, and the correlations between these electrical disorders are still valid and assertive.^12,13^ The association of these pathologies is further clarified when they are seen through the ECG/VCG dyad. (Figure 1)

Based on the abovementioned facts, during the last decade we were able to develop the performance of the binomial electrovectorcardiogram in the context of the most varied pathologies. This binomial can add sophistication to the already known clinical entities, in addition to a greater accuracy of the recent electrocardiographic definitions (such as Brugada, early repolarization, etc.)

Our experience, both academic and scientific, led us to join these new ECG/VCG acquisitions, and to open a window into the observation of the electrical phenomena of the heart. The literature has shown that the more sophisticated vectorcardiogram makes it easier for us to observe punctual phenomena that are not defined by the ECG.

### Limitations

Because there are only a few centers that are capable of performing routine vectorcardiography, there remains no doubt that this comparative study requires specific training, just like any other diagnostic method, through didactic bibliography and distance learning material. The purpose of this publication is guided by the acknowledgement of this situation.

### The electrovectorcardiography binomial

The experience with the VCG during these last decades shows the greater specificity and sensitivity of this method to detect the subtleties of these diagnoses. In comparison with the ECG, the VCG shows some advantages; however, when in association, they can help us differentiate between some very ordinary situations in clinical practice.

**Figure 2:**
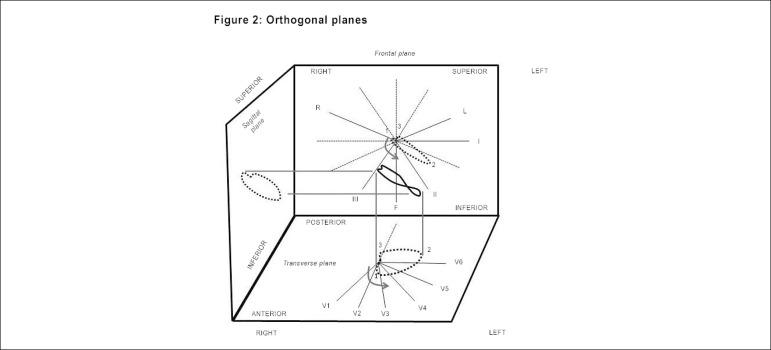
Electrical activation of the heart and its projection in the three spatial planes, thus giving origin to the vectorcardiographic loops in the respective planes.

The three-dimensional spatial orientation of both the atrial and the ventricular activity provides a far more complete observation tool than the linear form of the ECG. The modern analysis of the ECG and its respective VCG, simultaneously obtained by the recent technique called electro-vectorcardiogram (ECG/VCG), brought a significant gain for the differential diagnosis of some pathologies.^1,3,4,8,14,15^ (Figure 2)

**Figure 3:**
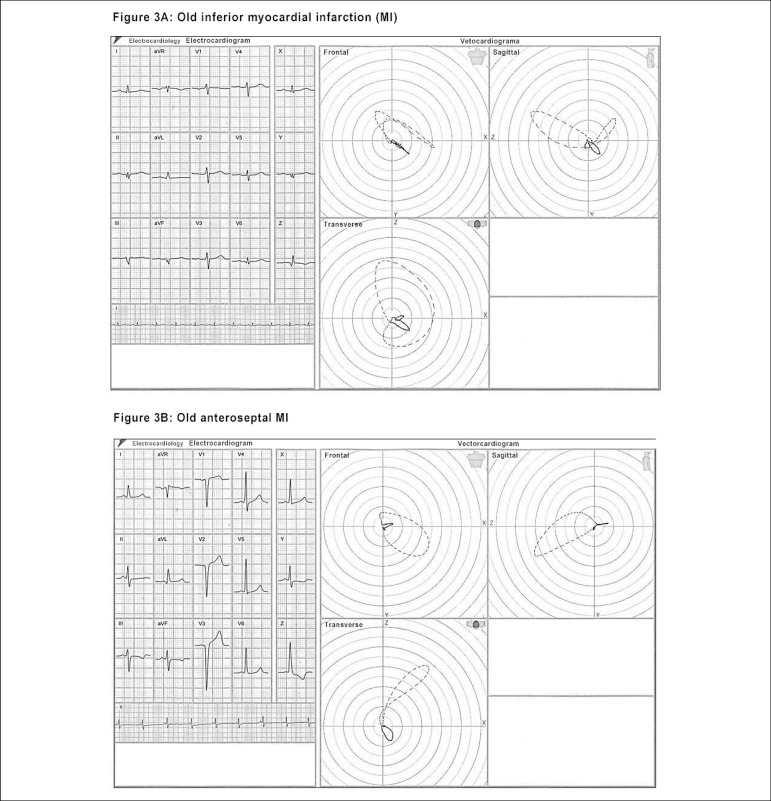
(A) Presence of myocardial infarction area in the inferior wall, with QRS vectorcardiographic loop onset oriented upwardly and to the left. (B) Aspect of a large myocardial infarction area from V1 to V6 and its respective loop in the transverse plane, with anomalous activation of the septum and exaggerated backward rotation, followed by deformation of the QRS loop.

The electro-vectorcardiographic analysis is very rich and consistent for the diagnosis of myocardial infarctions (MI), since the difficulties in defining pathological Q waves or the loss of R waves in the ECG can be very clearly visualized in the ECG/VCG. This association helps us to define the real changes in the direction and orientation of the vectorcardiographic loops created by the areas of myocardial infarction, in both the transverse and the frontal planes.^16-18^ (Figure 3)

**Figure 4:**
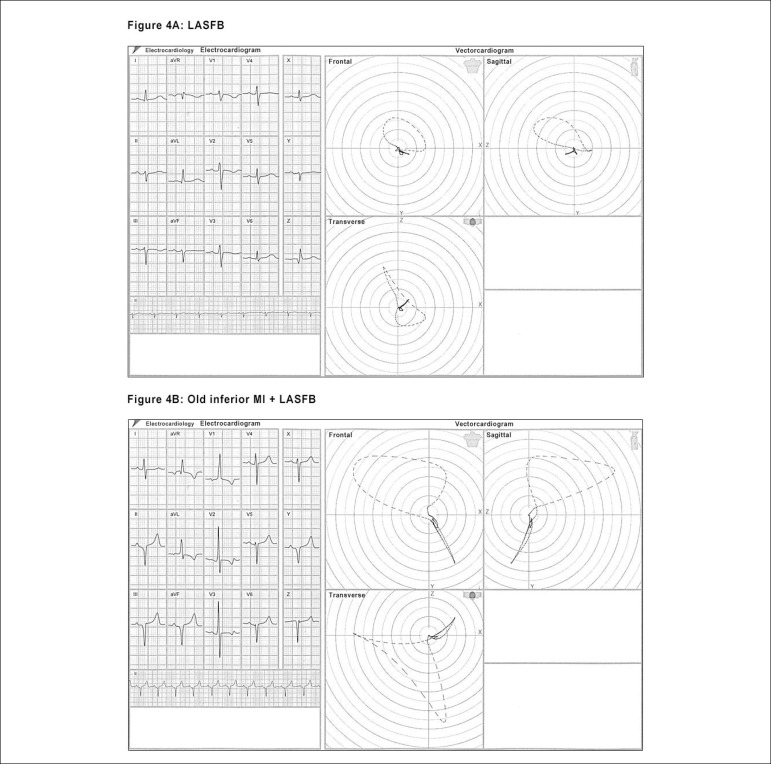
Aspects of the VCG loops in the Inferior MI (in Fig. 3A of the previous item, note the QRS loop onset upwardly in the frontal plane, with more than 30 ms duration [15 comets]); in Figure 4A – LASFB, observe the QRS loop onset downwardly (through the posterior-inferior division), with counterclockwise rotation and the major portion of the loop oriented upwardly and to the left; and in Figure 4B - LASFB + inferior MI, the association of the two entities; note that the QRS loop in the frontal plane is directed upwardly, with clockwise rotation, and after 30 ms it changes its orientation, with counterclockwise rotation characterizing the LASFB.

Another important differential aspect obtained by the electro-vectorcardiogram is the investigation of the presence of a myocardial infarction area in the inferior wall, of a left superior fascicular block, or the association of both pathologies. The association of MIs with the presence of fascicular or troncular blocks can be fully characterized by the ECG/VCG association. The inferior MIs with a left anterosuperior fascicular block (LASFB), and the anterior MIs with a right bundle-branch block (RBBB) are typical examples of the importance of the ECG/VCG association for a differential diagnosis.^19,20^ (Figures 3A and 4A and B)

**Figure 5:**
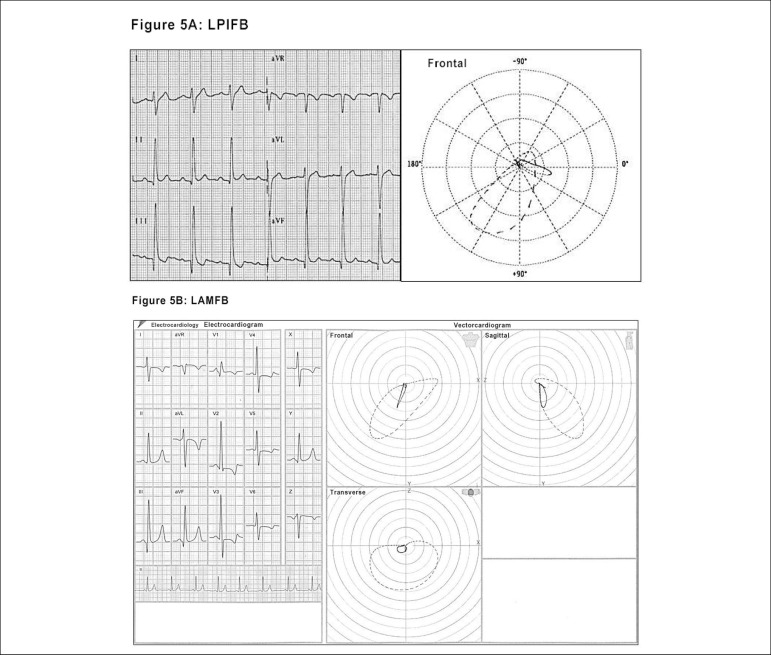
Electro-vectorcardiographic aspects of LASFB - Fig. 4A of the previous item, left posteroinferior fascicular block (LPIFB) - Figure 5A and left anteromedial fascicular block (LAMFB) - Figure 5B.

The spatial orientation of the fascicular blocks can be better understood through the electro-vectorcardiogram. The septal vector orientation and the direction of the vectorcardiographic loop activation neatly characterize the fascicular blocks and their associations through the ECG/VCG, since they define the electrical path of this phenomenon, thus characterizing exactly the position of the blocks.^21-24^ (Figures 4A and 5(A and B)

The VCG complements the ECG in the analysis of acute myocardial infarctions and makes the differential diagnosis of the associations with blocks and chamber hypertrophies. There is no doubt that the most ordinary situations in the cardiological routine, such as absent or exaggerated R waves from V1 to V3, require a more sophisticated definition in this region that depicts many expressions of different pathologies. Therefore, in this region the ECG/VCG is capable of characterizing the presence of: ^22-27^ (Figures 5B, 6[A, B, C and D])

**Figure 6:**
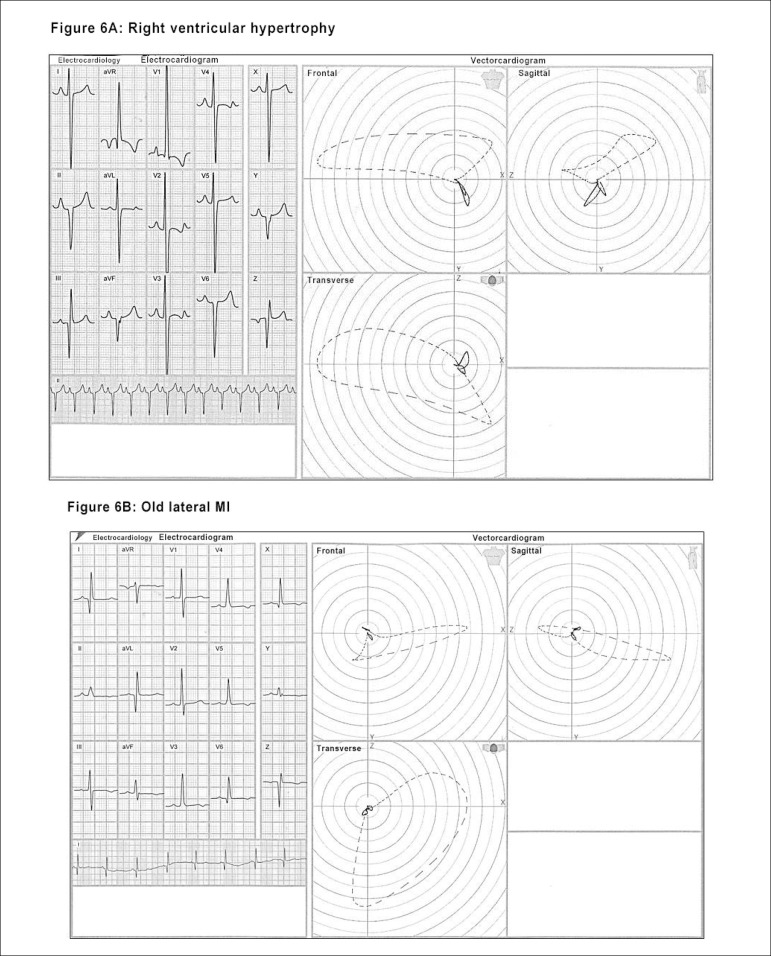
Illustrations of differential diagnoses identified by the ECG/VCG in different pathologies: left anteromedial fascicular block (LAMFB) - see Fig.5B; right ventricular hypertrophy (RVH) - Figure 6A; lateral infarction - Figure 6B.

**Figure 6:**
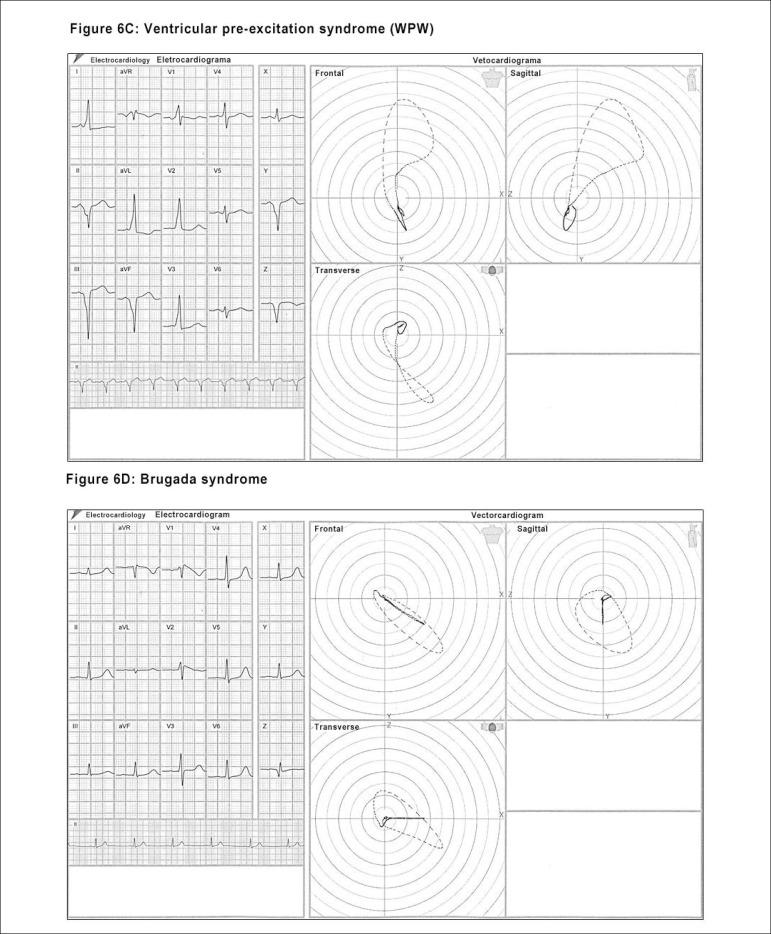
Illustrations of differential diagnoses identified by the ECG/VCG in different pathologies: left anteromedial fascicular block (LAMFB) - see Fig.5B; ventricular pre-excitation syndrome (WPW) - Figure 6C; Brugada syndrome - Figure 6D.

a) Left anteromedial fascicular block (LMFB)
b) Right ventricular hypertrophy (RVH)
c) Lateral infarction
d) Ventricular pre-excitation syndrome (WPW)
e) Brugada syndrome

**Figure 7:**
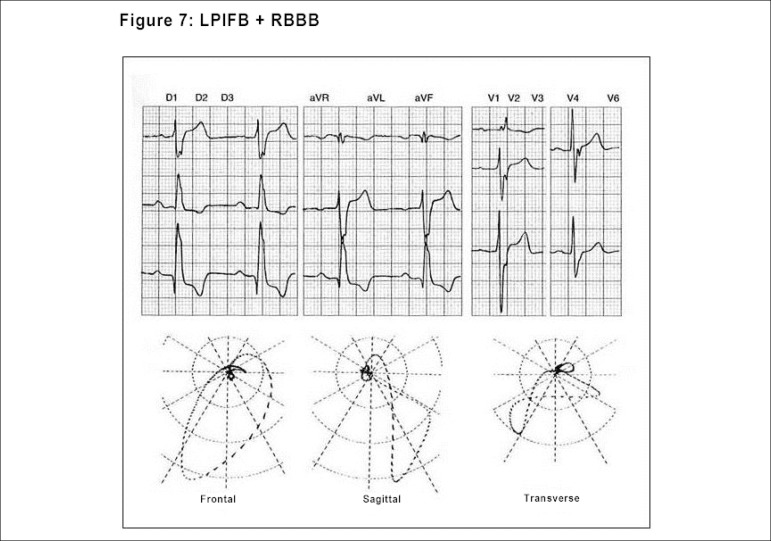
Vectorcardiographic aspects of the association of LPIFB and RBBB: axis to the right in the frontal plane (LPIFB), with most of the QRS loop in the frontal plane, oriented downwardly and to the left, and the QRS loop in the transverse plane, slowly ending forwardly and to the right (RBBB).

The ECG/VCG is the gold standard to identify complete and fascicular blocks, because it can differentiate them either in isolation or in association with other blocks. The electrical path marked by the ventricular activation loops can identify the blocks, as well as other associations.^21,23,28^ (Figure 7)

The end-conduction delays, previously denominated incomplete right bundle-branch blocks, are neatly defined by the ECG/VCG. These findings can be mistaken for the left phenomena and also can mimic a myocardial infarction area. Thus, the association of ECG/VCG solves the doubts that arise from the presence of these delays, which can be either the variants from the normal, or even suggest a conduction disorder in specific areas of the right ventricle.

**Figure 8:**
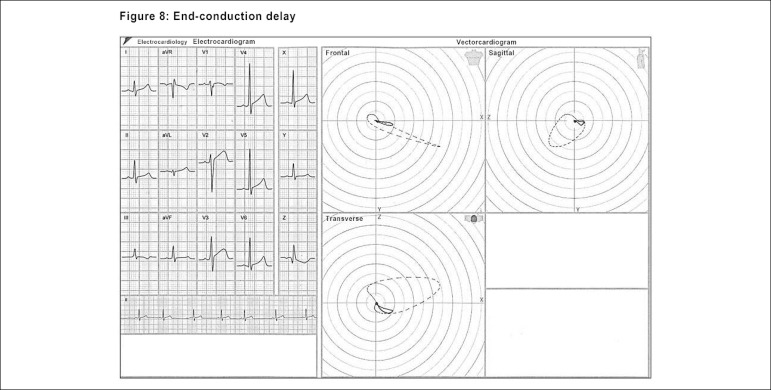
Electro-vectorcardiographic aspects of the end-conduction delay.

The presence of the end-conduction delay (ECD) is clarified in the ECG/VCG by the S_1_S_2_S_3_ pattern, with the S wave in D_2_ greater than the one in D_3_, qR in aVR and presence of S wave from V_1_ to V_6_. The ECG/VCG confirms the ECD position backwardly and to the right in the transverse plane, and upwardly and to the right in the frontal plane.^25^ (Figure 8)

**Figure 9:**
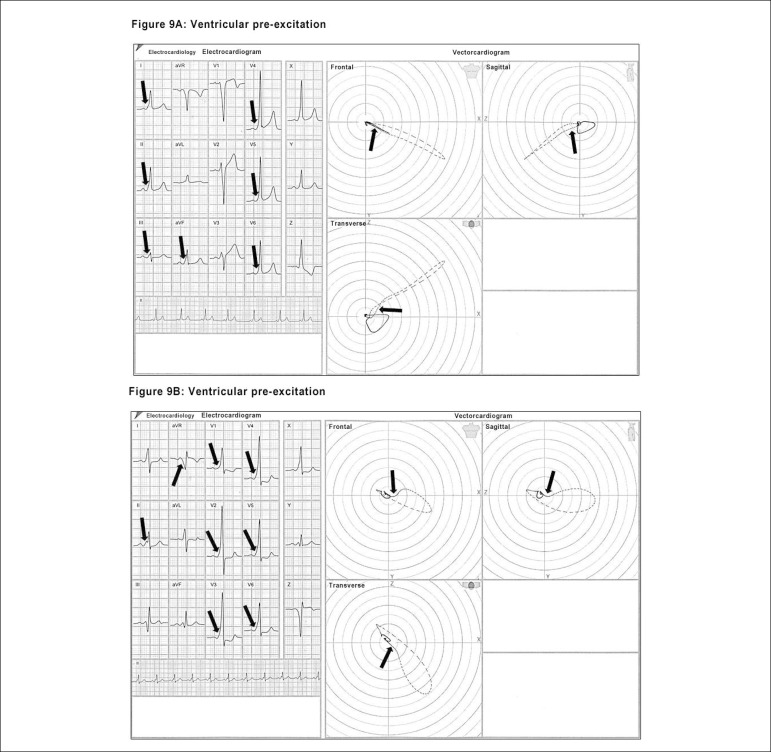
(A and B). Electro-vectorcardiographic aspects of the ventricular pre-excitation, highlighting the presence of the delta wave (arrows).

One of the definitive identifications obtained by the ECG/VCG association is that of the presence of a ventricular pre-excitation (WPW). The presence of the delta wave is very clearly seen in the beginning of the QRS loop by the proximity of the comets at the onset of the QRS loop, characterizing the delay caused by the accessory pathway, thus also establishing the position of the anomalous bundle at the valvular annulus.^26^ (Figure 9 [A and B])

One of the most recent diagnostic achievements of the ECG/VCG refers to the criteria to establish Brugada syndrome patterns, as well as the early repolarization (ER) phenomenon. It is important to emphasize that, in typical cases, there are no difficulties to make the electro-vectorcardiographic identification of both conditions. Due to the severity of the first, to the variability of the second between normal cases and other pathologies, and also to the possibility of having an early repolarization pattern located in a more anterior area, it became essential to make an adequate distinction between them. From the ECG/VCG viewpoint, there are no guidelines set to date to identify J-wave abnormalities. Specific ECG/VCG patterns of the J-wave abnormalities, namely the Brugada syndrome (BrS), and the early repolarization pattern (RP) were studied by our research team. An important qualitative and quantitative analysis of the ECG/VCG was carried out in all the study population, specifically regarding aspects of the area comprising the terminal portion of the QRS loop, the J point and the ST segment. This analysis showed a neat end-conduction delay (ECD) in all the individuals (in both the BrS and the RP groups). This ECD is characterized by a conduction delay greater than 10 ms at the final portion of the QRS in all the planes, either to the right or to the left (slowing of the comets, which tend to get closer and, eventually, to merge).

In the transverse plane, the QRS loops showed a counterclockwise rotation, with the ECD beginning posteriorly and ending anteriorly, with the main difference between the groups being the ECD position.

In BrS type 1 patients (Figure 6D) we see a counterclockwise rotation of the terminal QRS segment, J point and onset of the ST segment around the medial portion, resembling a "nose" in profile. The ECD position was in the right quadrant in all BrS patients and its duration was significantly longer. A greater than or equal to 30 ms ECD had 100% sensitivity and 77% specificity to diagnose Brugada syndrome. The BrS group showed a break at the end of the QRS loop right after the ECD, which resembled a "nose", right before the onset of the T-wave loop. This "nose" pattern was present in all the BrS type 1 patients.

**Figure 10:**
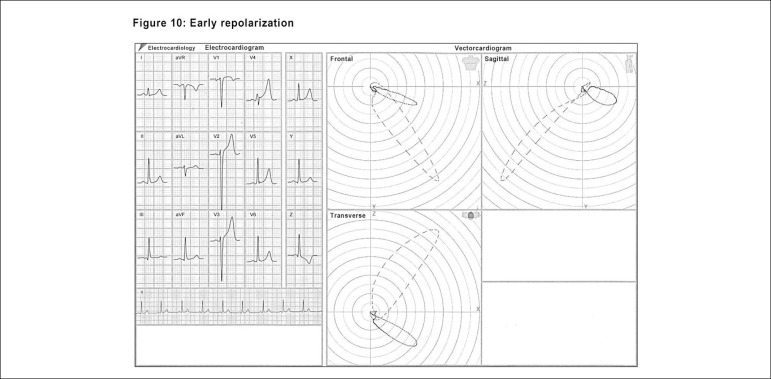
Characteristic electro-vectorcardiographic aspects of the early repolarization (ER) pattern: Transverse plane: QRS loop onset with counterclockwise rotation; the ECD began posteriorly and ended anteriorly and in the left quadrant in all ER patients. At the terminal segment of the QRS loop (now with clockwise rotation), 100% of the ER group showed, at the end of QRS loop, a “fishhook” pattern.

In the ER pattern^9,25^ (Figure 10) we see a clockwise rotation (terminal portion of the QRS) of the same segments, resembling a "fishhook". In all the ER cases the ECD position was in the left quadrant, with a shorter duration. In 100% of the ER patients, the final portion of the QRS loop showed a fishhook pattern.

Characteristic electro-vectorcardiographic aspects of the type 1 Brugada syndrome (see Figure 6D): transverse plane: QRS loop onset with counterclockwise rotation; ECD began posteriorly and ended anteriorly and in the right quadrant in all BrS patients; BrS showed a "break" at the end of the QRS loop after the ECD, in the terminal segment of the QRS loop, resembling a "nose", right before the T wave loop onset; this "nose" pattern was present in all type 1 BrS patients, but in none of ER patients.

**Figure 11:**
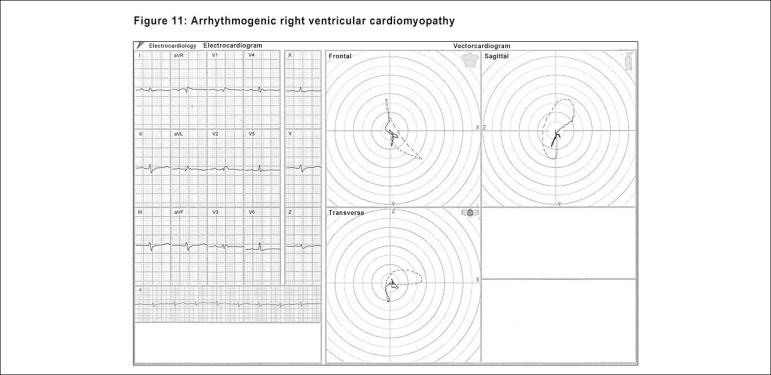
Electro-vectorcardiographic aspects found in ARVC, displaying the extremely long conduction delay at the end of the QRS loop.

A very important arrhythmogenic pathology, the arrhythmogenic right ventricle cardiomyopathy (ARVC), has an almost definitive assessment tool in the electro-vectorcardiographic diagnosis. The end-conduction delay with low voltage and long duration to the right (forward or slightly backwards) characterizes the phenomenon with great accuracy, with the differential diagnosis being very important, since this pathology may lead to severe arrhythmias. (Figure 11)

ARVC sometimes presents with an aspect similar to the RBBB, although with a very low voltage that is different from that block. It can also show an ECD aspect on the right and be slightly backwards. The presence of a negative T wave in V_1_, V_2_, V_3_ and left posteriorly located in the transverse plane of the ECG/VCG is crucial for an accurate diagnosis.

**Figure 12:**
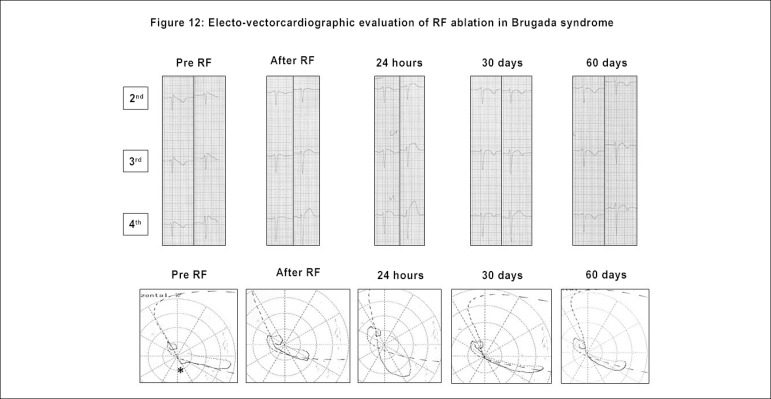
Evolution of the electro-vectorcardiographic aspects after radiofrequency ablation over time in a patient with ECG-type 1 pattern of Brugada syndrome.

The ECG/VCG has been used in the follow-up of the new arrhythmia ablation procedures, as in Brugada syndrome, where it cooperates in characterizing the successful cases and differentiating them from the unsuccessful procedures. The initial and already consecrated experience of the ECG/VCG in the ventricular pre-excitation and in the ablation procedures for this syndrome has brought subsidies for the recently described observations. The Brugada syndrome has shown to be very dynamic regarding its arrhythmogenic substrate, and the ECG/VCG follow-up can be very useful to define this process.^29,30^ (Figure 12).

## Appendix

For additional information, please see the online version of this article.
